# Editorial: Digging deeper: understanding root-pathogen interactions

**DOI:** 10.3389/fpls.2024.1549017

**Published:** 2025-01-10

**Authors:** Chun Sui, Frank L. W. Takken

**Affiliations:** ^1^ Institute of Medicinal Plant Development (IMPLAD), Chinese Academy of Medical Sciences & Peking Union Medical College (Key Laboratory of Bioactive Substances and Resources Utilization of Chinese Herbal Medicine, Ministry of Education & National Engineering Laboratory for Breeding of Endangered Medicinal Materials), Beijing, China; ^2^ Molecular Plant Pathology, Swammerdam Institute for Life Sciences, University of Amsterdam, Amsterdam, Netherlands

**Keywords:** root diseases, medicinal plant, metabolomics, PR proteins, fusarium wilt, rhizospheric microorganism

Root diseases caused by bacteria, fungi, oomycetes and viruses have a major impact on plant growth and agriculture. It is a great challenge to economically and environment-friendly control these soil-borne diseases. Various strategies to target specialized and/or generic pathogens have been developed, and although use of genetic resistance in cultivated crops is considered the most efficient and sustainable solution to counter root disease, such resistances are typically rare and quantitative in nature (Williamson-Benavides and Dhingra, 2021). Given this, it is essential to increase our understanding of the mechanisms underlying the occurrence of root diseases to aid development of novel prevention and control methods.

Current agricultural practices rely on the use of chemical pesticides to antagonize pathogens, among which the soil-borne ones that are the hardest to control. Especially for perennial crops, disease outbreaks are increasingly more frequent and severe with the change in global climate conditions resulting in more extreme weather conditions (Singh et al., 2023). The soil-borne nature of the pathogens makes chemical control often inefficient and not sustainable, as it also impacts beneficial microbes. How to break this vicious cycle?

The recent developments in molecular technology, especially “omics” techniques applied on host plant and microbiome, have accelerated our understanding of the molecular mechanisms underlying soil-borne disease pathogenesis and resistance. These insights provide a foundation for the development of novel strategies to control and/or prevent diseases. Grafting susceptible scions on a resistant rootstock confers protection against Fusarium wilt in tomato. Various resistance genes have been identified in tomato, but how these confer resistance to the fungus is currently unknown. In this Research Topic, Šimkovicová et al. used proteomics to identify the xylem sap proteins whose abundance correlates with disease resistance. Whereas the different immune receptors each induced a distinct set of pathogenesis-related proteins, four of these PR proteins were shared, making them prime candidates for restricting fungal proliferation. Besides PR proteins also cell wall fortifications play an important role in resistance against root invading pathogens. Clubroot resistance mediated by the CR genes *Rcr1* and *Crr1 rutb* in canola was shown to correlate with induced lignin accumulation around the infection sites, which agrees with transcriptomic studies that show induction of the phenylpropanoid pathway (Tu et al.). The rhizosphere microbiome provides another promising source for protection of plants against soil-borne diseases. Li et al. isolated a strain of *B. amyloliquefaciens*, BA-4, from the rhizosphere soil of healthy apple trees. Besides positive effects on plant growth the strain was found to exert a broad-spectrum antifungal activity against five crucial apple fungal pathogens. Metabolomics can be used to identify the probiotic compounds from the rhizosphere that selectively stimulate the growth and activity of beneficial microorganisms. Applied to tomato and other Solanaceae crops the identified metabolites provide protection of the plant to wilt disease (Wen et al., 2023). Specific bacteriophages combinations are another means to decrease the incidence of bacterial wilt diseases. A reduction of up to 80% can be achieved, exemplifying their potential as precision tools to control pathogenic bacteria (Wang et al., 2019). Another example of a molecular mechanism that is currently being explored to control diseases is based on virus induced gene silencing (VIGS). This technology is developed to not only control pests and eukaryotic pathogens, but also prokaryotic phytopathogens (Middleton et al., 2024). Viral vectors can induce production of small interfering RNAs (siRNAs) by the plant that are used by the RNA-induced silencing complex (RISC) to target mRNAs from the pathogen. A prerequisite for this strategy to control soil-borne prokaryotic phytopathogens is that the plant-derived siRNAs are transferred to the roots and/or secreted into the rhizosphere to interfere with growth and reproduction of the pathogen. Jang et al. demonstrate the effective use of plant-induced bacterial gene silencing against *Ralstonia pseudosolanacearum.* Tobacco Rattle Virus-mediated gene silencing was successfully used to control bacterial wilt symptoms in *Nicotiana benthamiana*. This technology provides a powerful tool for the identification of novel bacterial virulence factors and holds great promise for application in the field.

Medicinal plants are a major source for the production of natural drugs. Many active ingredients in drugs and potions are produced from the roots of medicinal plants. Besides decreasing yield, root diseases exert adverse impact on the merchandise properties. The pathogen, or toxins produced by pathogens, may affect the biosynthesis of metabolites with medicinal value. In the comprehensive manuscript by Sui et al. recent research progress on root rot in medicinal plants is reviewed, providing leads for a better control of these pathogens. Excitingly, some special metabolites synthesized by roots of medicinal plants or associated rhizospheric microorganism have unexpected potential to antagonize pathogens (Compant et al., 2025). The microbiomes in the bulk soil, rhizosphere and endosphere are significantly different and they form an intricate network in which the microbiome contributes to the biosynthesis of metabolites with medical value (Zhang et al.).

Soil-borne diseases will remain a threat to agricultural production and can impact the quality and safety of our food, feed and pharmaceuticals. This Research Topic, focusing on below ground plant-pathogen interactions, provides new leads to develop strategies to prevent and control root diseases. These non-pesticide based strategies contribute to human health and well-being by improving crop quality and enhancing ecological environment.
